# Case of Third Dentition

**Published:** 1850-01

**Authors:** A. Berry

**Affiliations:** Raymond, Miss.


					﻿For the Dental Register of the West,
[CASE OF THIRD DENTITION.
Messrs. Editors.—I met to-day with a case of third den-
tition, which may be interesting to some of your readers.
A negro boy, belonging to Dr. W. S. J. of this vicinity
about six years of age, was attacked with sickness, atu
with obstinate constipation of the bowels, and trea' i
exhibition of large and repeated doses of calor '
hundred grains had been given without produci .
effect. Blistering the spinal regions, castor o . oi ano sp . . !
turpentine, enemas, and other remedies were ’	: rr
but these, as well as the calomel, proving in
the torpidity of the bowels, further medication . /	'■
to, as the case was considered hopeless. E
supervened. His mouth and gums were d'	co
period, and his teeth, including the permar.’;
had all made their appearance, were chang • ,	■
white to a dark color. Last year, when ah. ,t u -■■■■
old, he lost the incisors in the course of a few w' &s, o
were soon succeeded by others—the full number cf infen c .?
and three superiors—one of the laterals being wanti: . ■ ■ .nch
from present appearances, it seems will not be renewed i
process of second dentition is still going on, but later than usual,
the anterior bicuspidati having all recently come through the
gums. The boy has been generally healthy since his sickness,
but is small for his age. His teeth of third dentition are firm
in their sockets, and look well.
A. BERRY, D. D. S.
Raymond, Miss., Nov. 23, 1849.
				

